# High‐Performance PbSe Quantum Dots with Palmitoyl Chloride and Their Application to Short‐Wavelength Infrared Photodetector Devices

**DOI:** 10.1002/smtd.202402237

**Published:** 2025-03-12

**Authors:** Haewoon Seo, Ah Young Lee, Eun Hye Lee, Dong Won Kim, Hyo Jin Hwang, Sunghoon Kim, Jong H. Kim, Sang‐Wook Kim

**Affiliations:** ^1^ Department of Molecular Science and Technology Ajou University Suwon 16499 Republic of Korea; ^2^ AI‐Superconvergence KIURI Translational Research Center Ajou University Suwon 16499 Republic of Korea; ^3^ Department of Applied Chemistry Dong‐Eui University Busan 47340 Republic of Korea

**Keywords:** halide passivation, PbSe quantum dots, photodetector, quantum dots, SWIR

## Abstract

Quantum dots (QDs), particularly those in the short‐wavelength infrared (SWIR) range, have garnered significant attention for their unique optical and electrical properties resulting from 3D quantum confinement. Among the various chalcogenide‐based QDs, lead chalcogenides, such as PbS and PbSe, are extensively studied for infrared photodetection applications. While PbSe QDs offer advantages over PbS, including a narrower bandgap and higher carrier mobility, they suffer from stability issues due to surface oxidation and particle aggregation. Conventional synthesis methods require additional post‐synthesis treatments for surface passivation with halides, which complicates the process. In this work, a novel synthesis approach that incorporates palmitoyl chloride (PalCl) into the traditional PbSe QD synthesis is introduced, effectively passivating the surface with Cl^−^ ions during the synthesis process. This method not only enhances the optical performance by producing a sharp exciton peak and allowing precise tuning of the absorption spectrum from 1100 to 1900 nm but also significantly improves the stability of the QDs in solution. The resulting QDs are successfully integrated into SWIR photodetectors (PDs), demonstrating exceptional specific detectivity of 1.08 × 10^12^ Jones at 1460 nm. This achievement draws great potential of the proposed synthetic method for advancing infrared optoelectronic devices.

## Graphical abstract

Comparison data of the absorption spectra of PbSe quantum dots with and without palmitoyl chloride (PalCl) treatment, and the external quantum efficiency (EQE) spectrum of a photodetector device based on PalCl‐treated quantum dots.

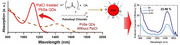

## 1 Introduction

Quantum dots (QDs), 0D semiconductor nanomaterials, exhibit distinctive properties due to 3D quantum confinement effects. These properties enhance optical and electrical performance across a wide spectral range, including ultraviolet (UV),^[^ ^1^, ^2^ ^]^ visible,^[^ ^3^, ^4^ ^]^ near‐infrared (NIR),^[^ ^5^, ^6^ ^]^ and short‐wavelength infrared (SWIR) wavelengths.^[^ ^7^, ^8^ ^]^ These characteristics have precipitated significant research interest in both the academic and industrial sectors for potential applications across various fields. Among emerging research areas, the development of SWIR‐sensitive quantum dot‐based photodetectors represents a promising frontier. The SWIR spectrum range, extending from 1–2 µm, offers superior penetration and reduced susceptibility to environmental interference compared to visible and NIR wavelengths.^[^ ^9^, ^10^ ^]^ This range is particularly less affected by atmospheric water vapor and solar irradiance, positioning it as a pivotal technology in the advancement of optical communications and imaging sensors.

In the SWIR spectrum, QDs predominantly consist of chalcogenide elements, with a diversity of compounds including lead chalcogenides (PbS, PbSe),^[^ ^11^, ^12^ ^]^ silver chalcogenides (Ag_2_Se, Ag_2_Te),^[^ ^13^, ^14^ ^]^ mercury telluride (HgTe),^[^ ^15^, ^16^ ^]^ and ternary compounds like copper indium selenide (CuInSe_2_).^[^ ^17^ ^]^ Among these, lead chalcogenides are the most widely employed. As a prototypical Group II‐IV QDs material, the lead chalcogenide series has established itself as the dominant material in the realm of QD‐based infrared photodetection. Since the demonstration of a photodetector using PbS within the 1300 nm spectral region by the Sargent group in 2004,^[^ ^18^ ^]^ lead chalcogenide QDs, characteristic of the Group II‐IV compounds, have emerged as the predominant materials in the development of quantum dot‐based infrared photodetection technologies.

Following the Sargent group's work, extensive investigations have been conducted on photodetectors based on PbS QDs, which are known for their exemplary performance derived from rigorous optimization and research.^[^ ^19^ ^]^ Nevertheless, to extend the photodetection capabilities into wavelengths beyond 1400 nm with PbS QDs, it becomes necessary to enlarge the particle size beyond 5 nm. This enlargement exposes unpassivated crystal facets, resulting in decreased stability, which represents a significant limitation of larger PbS QDs. Consequently, there has been an increasing interest in exploring materials with bandgaps narrower than those of PbS QDs to overcome these challenges.

PbSe, like PbS, is categorized within the lead chalcogenide group. It possesses a narrower bulk bandgap (PbS: 0.42 eV, PbSe: 0.26 eV)^[^ ^20^, ^21^ ^]^ and a significantly larger Bohr radius (PbS: 20 nm, PbSe: 46 nm),^[^ ^22^ ^]^ thereby facilitating more pronounced quantum confinement effects. In its bulk crystalline state, PbSe demonstrates substantially enhanced carrier mobility relative to PbS.^[^ ^23^ ^]^ These properties confer distinct functional advantages to PbSe in a variety of optoelectronic applications over PbS. However, PbSe suffers from an inherently unstable surface that is extremely prone to oxidation,^[^ ^24^ ^]^ and its synthesis process is characterized by a strong propensity for particle aggregation.^[^ ^25^ ^]^ Consequently, precise optimization of the synthesis protocols is imperative to mitigate these issues and enhance the material's stability and performance.

Typically, the synthesis of PbSe involves using a lead precursor and oleic acid (OA). However, PbSe is susceptible to oxidation due to decarboxylation,^[^ ^26^ ^]^ which compromises its optical stability, necessitating additional surface treatment using halides. An alternative synthesis method that does not use OA primarily employs a lead chloride (PbCl_2_) precursor with oleylamine (OLA), but this approach requires ligand exchange with OA to ensure solvent dispersion and stability.^[^ ^27^, ^28^ ^]^ An alternative method involves the synthesis of QDs using conventional Pb precursors and OA, followed by surface stabilization with Cl^−^ ions using ammonium chloride (NH_4_Cl)^[^ ^29^ ^]^ or PbCl_2_.^[^ ^30^ ^]^ Another approach is the synthesis of PbSe QDs via cation exchange after the initial synthesis of CdSe or ZnSe QDs, utilizing PbCl_2_ for the cation exchange process.^[^ ^31^, ^32^ ^]^ PbSe synthesized using the aforementioned methods exhibited very stable optical performance beyond 1400 nm due to the effects of Cl^−^ surface treatment and the device operation results were also very impressive. These results indicate that Cl^−^ surface treatment is very effective for PbSe QD. However, these methods present the drawback of necessitating additional post‐synthesis processing steps. Particularly, the cation exchange method requires the synthesis of substantially large CdSe or ZnSe cores to achieve wavelengths beyond 1500 nm. Therefore, new methods to overcome these limitations are required.

In this work, we demonstrate a synthesis method for PbSe QD with superior performance by incorporating palmitoyl chloride (PalCl) into the conventional synthesis process. When comparing the results before and after the addition of PalCl, we observed that the PbSe QD with PalCl exhibited a blueshift of the 1st exciton peak in the absorption spectrum, and this peak appeared very sharp. To elucidate the underlying mechanism for the effect, various analysis was conducted, including Fourier‐transform infrared spectroscopy (FT‐IR), nuclear magnetic resonance (NMR), and mass spectrometry, confirming that the observed effects were due to Cl^−^ ion on the PbSe surface. Leveraging these insights, we managed to finely tune the absorption spectrum from 1100 to 1900 nm by optimizing the growth temperature and precursor concentration, while maintaining a narrow absorption shoulder. Furthermore, we observed a substantial improvement in stability in solutions. Afterward, we successfully implemented a SWIR photodetector in a photodiode‐based device, achieving performance at 1460 nm with an external quantum efficiency (EQE) of 8.93% and specific detectivity (D^*^) of 1.08 × 10¹^2^ Jones at 0 V, and an EQE of 23.96% and D^*^ of 4.08 × 10¹⁰ Jones at 0.5 V.

## 2 Results and Discussion

As previously mentioned, we employed PalCl to facilitate the donation of Cl^−^ during the synthesis process. PbSe QDs were synthesized by a slightly modified method based on a previously reported procedure.^[^ ^33^ ^]^ Initially, lead acetate (Pb(oAc)_2_) and oleic acid (OA) were mixed in 1‐octadecene (ODE) solvent to form Pb‐oleate. Subsequently, tris(trimethylsilyl)selenide (TMS‐Se) was injected to synthesize the PbSe QD. During this process, the proportion of OA was gradually reduced and replaced with an equivalent proportion of PalCl.

**Figure 1:**
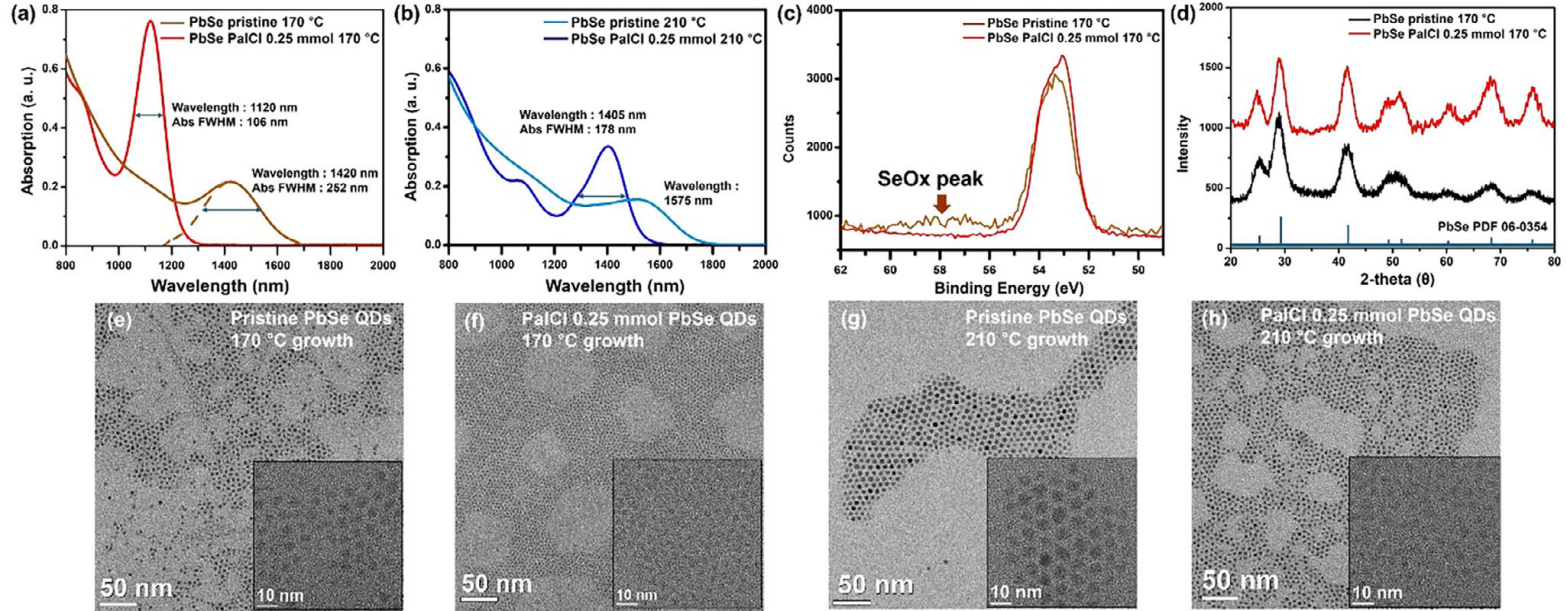
Comparison data of PbSe QDs with and without PalCl treatment. a) Absorption spectrum at 170 °C b) Absorption spectrum at 210 °C c) XPS spectrum for Se 3d d) XRD patterns. e) TEM images of pristine PbSe QDs at 170 °C f) PalCl treated PbSe QDs at 170 °C g) TEM images of pristine PbSe QDs at 210 °C h) PalCl treated PbSe QDs at 210 °C.

Initially, 0.25 mmol of PalCl was added for the synthesis, and the results are presented in **Figure** **1**. (OA: 4.5 mmol) Figure 1a illustrates the absorption spectra of PbSe QDs with and without the addition of PalCl during the synthesis process. When the growth temperature was set at 170 °C, the PbSe QDs with 0.25 mmol of PalCl exhibited a significantly blueshifted absorption peak compared to pristine PbSe QDs synthesized under the same conditions, with a very sharp first exciton peak (pristine: 1420 nm, FWHM: 252 nm; PalCl 0.25 mmol: 1120 nm, FWHM: 106 nm). Additionally, the absorption intensity at the peak was slightly higher. Figure 1b presents the absorption spectra at a growth temperature of 210 °C, showing a similar trend as observed in Figure 1a. At 210 °C, the FWHM of PalCl‐treated PbSe QDs was slightly broadened but still exhibited superior absorption performance compared to pristine QDs under the same conditions. The peak position of the PalCl‐treated PbSe QDs was observed at 1405 nm, close to the peak position of the pristine PbSe QDs in Figure 1a, and still showed a higher absorption coefficient. This phenomenon is presumed to be caused by the participation of Cl^−^, which is generated by the decomposition of PalCl in the initial stages of synthesis. The sharp absorption peak indicates that the very strong x‐type ligand, Cl^−^, binds tightly to the quantum dot surface, regulating the growth process and resulting in a very narrow particle size distribution. Simultaneously, Cl^−^ also plays a role in preventing oxidation, leading to higher peak absorption.^[^ ^29^ ^]^ Separately, there have been reports that halide passivation significantly reduces the trap density of PbSe quantum dots.^[^ ^34^ ^]^ Based on these findings, we believe that halide surface treatment using PalCl not only regulates the size distribution of PbSe quantum dots but also improves their absorption performance through strong surface passivation. When measuring the X‐ray Photoelectron Spectroscopy(XPS) of pristine QDs and QDs with PalCl as shown in Figure 1c, the Se 3d peak was similar for both, but the pristine PbSe showed a slight SeOx peak in the 56–60 eV range, which indicated the oxidation of PbSe surface. It is not clear whether this partial oxidation of Se occurred during the synthesis process or the purification process, but it indicates that Cl^−^ derived from PalCl is effective in protecting the QD surface. Subsequent X‐ray Diffraction (XRD) measurements of the two samples revealed that while it is unclear whether the improved crystallinity is due to the enhanced size distribution from the PalCl treatment or the protective effect of Cl⁻, the PalCl‐treated PbSe QDs exhibited higher crystallinity compared to the pristine PbSe QDs (Figure 1d).

To confirm that the narrow FWHM observed in Figure 1a,b is based on the size distribution, we performed TEM measurements. In both cases, it was observed that the PbSe with PalCl had a superior size distribution compared to pristine, and the particle sizes were relatively smaller. Figure 1e shows the TEM image of pristine PbSe QDs, which exhibit a size distribution ranging from ≈3 to 5 nm, although some very small particles are also observed. In contrast, in Figure 1f, the PbSe QDs treated with PalCl have a reduced particle size in the range of 2–3 nm, with significantly less size variation. This phenomenon is also evident when compared with the TEM image of high‐temperature grown PbSe QDs in Figure 1b.(Figure 1g,h) Additionally, the size distribution, based on the inset TEM images of Figure 1e–h, shows that the size distribution of the PalCl‐treated QDs is remarkably uniform (see Figure S1, Supporting Information). For pristine PbSe QDs, the size distribution was 4.1 ± 1.3 nm under the 170 °C growth condition and 4.6 ± 2 nm under the 210 °C condition. In contrast, with the addition of 0.25 mmol PalCl, the size distribution was 2.8 ± 0.5 nm and 3.9 ± 0.9 nm under the same growth conditions, respectively. Overall, the QDs synthesized with PalCl exhibited a more uniform size distribution, with a notably smaller size deviation under the 170 °C condition, even though the distribution curve for pristine PbSe QDs was less precise. To further clarify this observation, the sample was taken immediately after TMS‐Se injection, and absorbance was measured to investigate the initial formation state of PbSe QDs. The absorption data showed that the samples with PalCl exhibited a blueshifted absorption peak, and a more pronounced absorption shoulder compared to the pristine samples (Figure S2, Supporting Information). Furthermore, to verify the actual presence of Cl^−^ on the surface of PbSe QDs with PalCl, X‐ray fluorescence (XRF) measurements were conducted, confirming the presence of Cl⁻ (Figure S3a, Supporting Information). Additionally, XPS also revealed peaks attributed to Cl⁻ (Figure S3b, Supporting Information).

As mentioned earlier, the role of PalCl lies in its ability to dissolve well in organic solvents, allowing Cl^−^ to be delivered in a uniform phase, which is believed to result in the excellent performance observed in Figure 1. This implies that substances like NH₄Cl or PbCl₂, which do not dissolve well in organic solvents, cannot be expected to achieve the same effect as PalCl. To address the points raised, we explored alternative precursors to PalCl as Cl^−^ ion sources and compared the results with our findings. The precursor used was NH₄Cl, and under identical experimental conditions with the same amount as PalCl, the absorption of PbSe quantum dots was found to be similar to that of the pristine state.(Figure S4a, Supporting Information) Notably, during the synthesis process, the transparent ODE solution turned pale yellow, which we believe resulted from the poor solubility of the inorganic NH₄Cl in the ODE solution. This phenomenon is also expected to occur with PbCl₂. To further clarify the results, we conducted experiments by adding various aliphatic chain compounds with similar functional groups to palmitoyl chloride (e.g., decanoic chloride, acetyl chloride) during the synthesis process.(Figure S4b, Supporting Information) The comparison revealed that as the chain length decreased, the effectiveness of Cl⁻ diminished, leading to the absorption of characteristics closer to those of the pristine state. Acetyl chloride exhibited negligible performance. These findings support our assertion that the addition of organic halides induces more uniform reactions, making them more effective in improving the optical performance of PbSe. This also explains why previous studies on Cl^−^ passivation opted for post‐synthesis treatment rather than introducing Cl^−^ sources during the synthesis process.^[^ ^29^, ^30^ ^]^

**Scheme 1:**
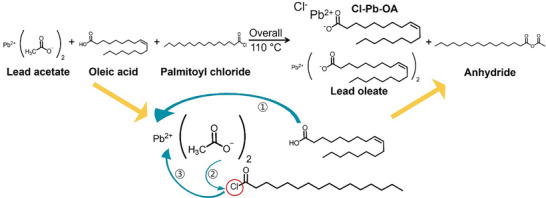
Schematic diagram of the chemical reaction mechanism occurring during the formation of Pb‐oleate with the addition of PalCl.

Regarding the observed change in the absorption peak of PbSe quantum dots with PalCl, we assumed that Cl^−^ ion by decomposition of PalCl was the main cause and hypothesized the mechanism in **Scheme** **1**. We aimed to validate this hypothesis through FT‐IR, ^1^H‐NMR, and mass spectrometry analyses. Scheme 1 illustrates the anticipated chemical reaction pathway when a small amount of PalCl is introduced during the formation of Pb‐oleate from Pb(OAc)_2_ and OA. In conventional Pb‐chalcogenide QD synthesis using Pb(OAc)_2_, excess OA is introduced to replace acetate, forming Pb‐oleate. The replaced acetate is converted to acetic acid, which is subsequently removed during the pre‐treatment process of the precursors. In this process, even in the presence of PalCl, the carboxylic acid group of OA attacks the Pb cation, and the acetate group of lead acetate detached, which is the same as the original process (step‐1). The detached acetate anion attacks the carbonyl group of PalCl (step‐2), and the Cl^−^ ion is detached by the SN‐2 reaction and attached to the Pb cation (step‐3), forming the PbCl(OA) precursor. In the process, PalCl accelerates the detached process of acetate compared with the no‐PalCl process. Subsequently, when the Se precursor is introduced, the synthesis proceeds to form PbSe QDs. As a byproduct of this reaction, acetic palmitic anhydride is formed.

This hypothesis is based on our observation that when only Pb(OAc)_2_ and OA are mixed in a solvent at room temperature, no change occurs. However, the addition of a small amount of PalCl causes the solution to become opaque. (Figure S5, Supporting Information) We infer that the resulting opaque solute is a PbCl(OA) precursor. To validate the hypothesis, we mixed Pb(OAc)_2_, OA, and a small amount of PalCl in hexane and conducted the analyses of the solution using FT‐IR, ^1^H‐NMR, and FAB‐Mass spectrometry. Figure S6a (Supporting Information) shows the FT‐IR spectrum of the solution, where a large peak was observed at 1710 cm^−1^ corresponding to the C═O stretching of OA. Typically, peaks appearing above 1800 cm^−1^ are associated with the symmetric stretch of the anhydrides. Upon closer inspection of the spectrum, peaks corresponding to the two C═O stretches of anhydrides were observed at 1820 and 1752 cm^−1^. To confirm the existence of anhydrides, ^1^H‐NMR was measured, and the presence of anhydrides was confirmed in the 2.0–2.5 ppm range.(Figure S6b, Supporting Information)

In the ^1^H‐NMR spectrum, we observe a singlet peak at 2.21 ppm and a triplet peak at 2.45 ppm. Typically, the hydrogen atoms adjacent to the carbonyl group in acetyl anhydride appear around the 2.2 ppm region, while for anhydrides with longer alkyl chains, a triplet peak is observed in the 2.4–2.5 ppm region. Based on this observation, we can infer that the structure of the anhydride present in the sample comprises an acetyl group linked to a palmityl group. The triplet peak at 2.45 ppm appears to be a superposition of two triplet peaks, indicating the presence of two different long‐chain anhydrides (Figure S7, Supporting Information). One of these is the previously mentioned acetyl palmityl anhydride, and the other is an anhydride formed from palmityl and oleic acid (OA). The anhydride formed from palmityl and OA is inferred to appear upfield due to its relatively higher electron density, and the peak is more pronounced due to the increased number of their β‐hydrogens.

Additionally, we performed ^13^C‐NMR analysis on the aforementioned sample, and the results confirmed the presence of peaks corresponding to the carbonyl groups of acetylpalmityl anhydride (168, 177 ppm) (Figure S8, Supporting Information). To definitively verify the structure of the anhydride, we conducted FAB‐Mass spectrometry analysis.(Figure S9, Supporting Information) Based on the two anhydrides discussed earlier, the molecular weight of acetylpalmityl anhydride is 298 m z^−1^, and that of oleylpalmityl anhydride is 520 m z^−1^. The mass spectrum reveals mass peaks corresponding to these two anhydrides. Additionally, we observed a mass peak at 524 m z^−1^, corresponding to the molecular weight of Cl^−^ bound Pb‐oleate. These findings corroborate the presence of the anhydrides and the Cl^−^ bound Pb precursor as hypothesized in Scheme 1.

**Figure 2:**
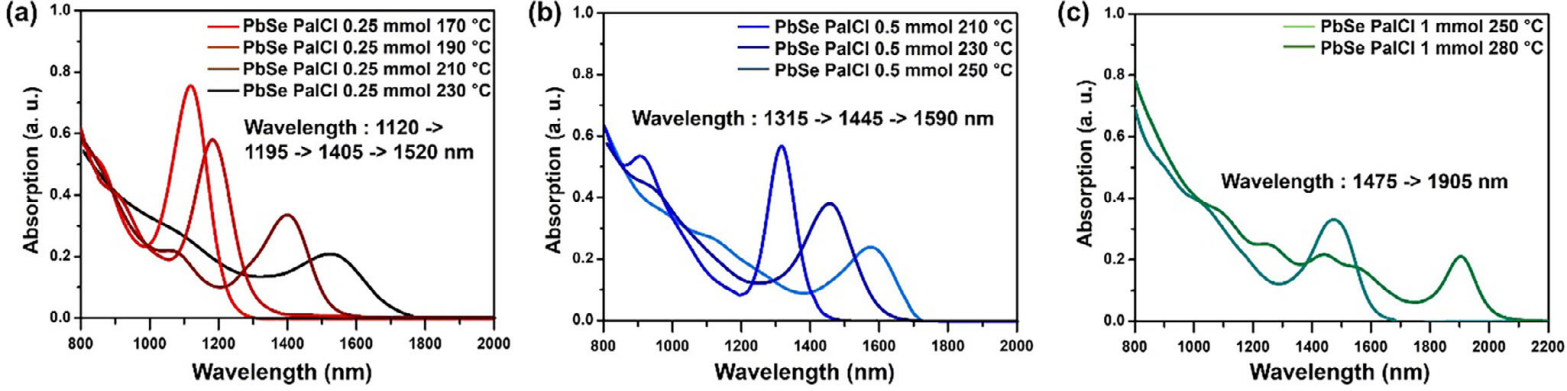
Absorption spectra of PalCl treated PbSe QDs with varying amounts of PalCl and growth temperatures. a) PalCl 0.25 mmol added, b) PalCl 0.5 mmol added, c) PalCl 1 mmol added. Analysis data of Pb‐oleate with the addition of 0.25 mmol of PalCl.

Based on the previous findings, we aimed to extend the absorption range of PalCl‐treated PbSe QDs by adjusting the growth temperature. As shown in **Figure** **2**a, with the addition of 0.25 mmol of PalCl, the absorption shoulder maintained a relatively sharp shape up to a growth temperature of 210 °C. However, at 230 °C, a broader peak began to emerge, and at 240 °C, the peak shape became significantly broader (Figure S10a, Supporting Information). TEM image of the sample at 240 °C revealed broad size variation and a tendency for the particles to start agglomerating (Figure S10b, Supporting Information). This indicates that particle ripening begins at 240 °C, suggesting that 210 °C is the optimal growth temperature for 0.25 mmol of PalCl.

Figure 2b presents the absorption spectrum of PbSe QDs with double the amount of PalCl (0.5 mmol, OA: 4.2 mmol). When retained at the optimal growth temperature of 210 °C, the absorption peak exhibited a blueshift to 1315 nm with a very sharp absorption shoulder. Increasing the growth temperature to 230 and 250 °C extended the absorption range to 1445 and 1590 nm, respectively. Further increasing the amount of PalCl to 1 mmol (OA: 3.8 mmol) and conducting the synthesis at 280 °C resulted in an absorption peak extending to 1905 nm.(Figure 2c) However, with 1 mmol of PalCl, the dispersion stability in solvents such as hexane and tetrachloroethylene showed a slight decline. As shown in Figure 2, increasing the amount of PalCl leads to a corresponding increase in the concentration of Cl^−^ ions. Under identical growth temperatures, a higher amount of PalCl results in a blueshift of the absorption peak position. Additionally, the presence of a higher concentration of Cl^−^ ions helps maintain the sharpness of the absorption peak even under elevated growth temperature conditions. These findings indicate that Cl^−^ ions play a significant role in controlling the growth of PbSe QDs.

**Figure 3:**
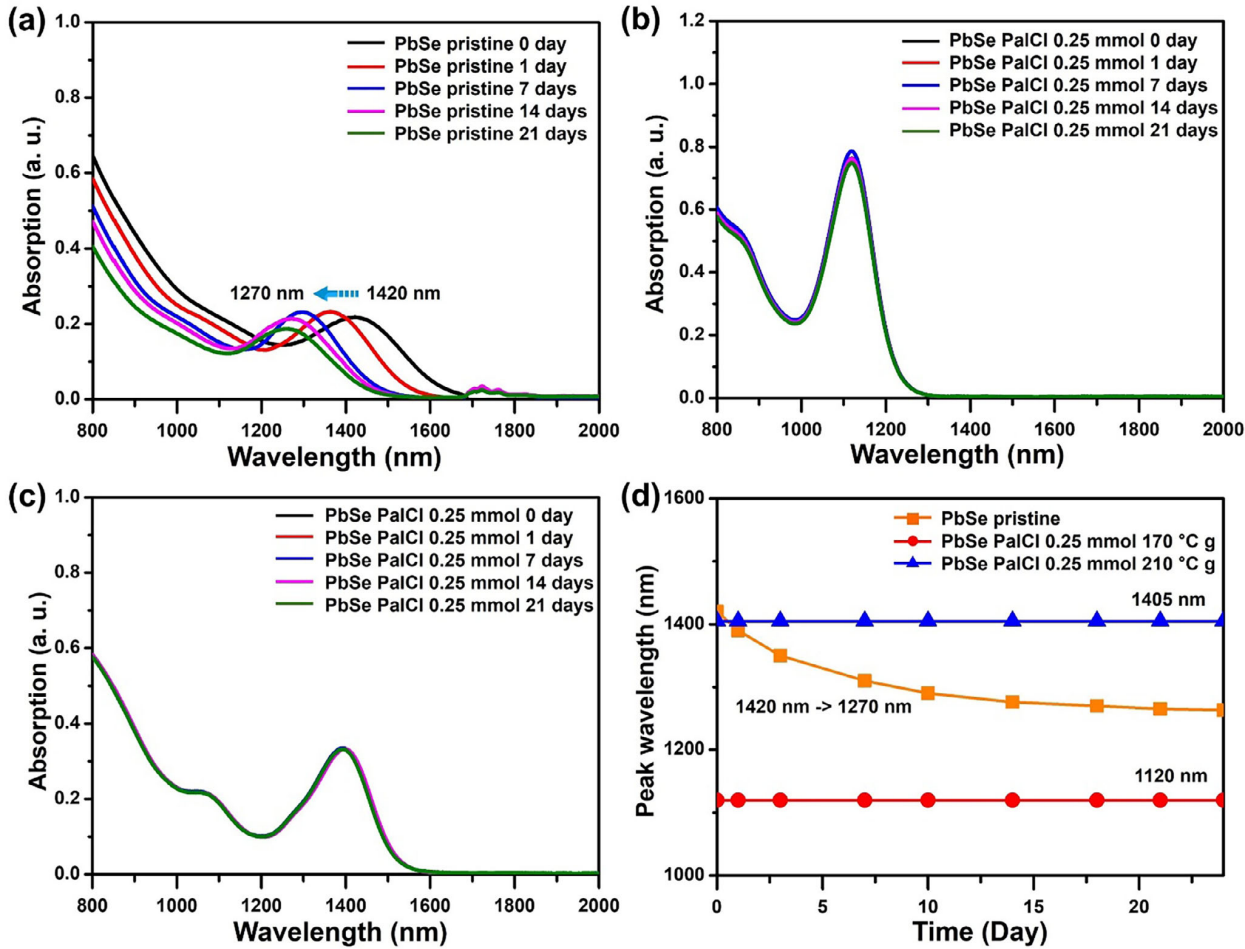
Time‐dependent changes in the absorption spectrum of PbSe QDs. a) Pristine PbSe QDs growth at 170 °C. 0.25 mmol of PalCl treated PbSe QDs, b) growth at 170 °C, c) growth at 210 °C. d) Plot showing the changes in the absorption peaks of the three types of QDs.

We conducted stability tests using an absorption spectrum analysis over time to evaluate the actual surface protection efficacy of Cl^−^. **Figure** **3** presents the test results over time for pristine PbSe QDs and PbSe QDs with 0.25 mmol of PalCl, both dispersed in TCE solutions. Figure 3a illustrates the temporal changes in the absorption wavelength of pristine PbSe QDs, showing a blueshift in the absorption peak as time progresses. The initial peak position at 1420 nm shifted to 1270 nm after 21 days, a phenomenon attributed to the oxidation of the PbSe quantum dot surface.^[^ ^24^ ^]^ In contrast, PbSe QDs synthesized with 0.25 mmol of PalCl maintained the same absorption peak through 21 days, indicating effective surface passivation by Cl^−^.

Figure 3b illustrates that PbSe QDs with 0.25 mmol of PalCl, growth at 170 °C, retained their original wavelength of 1120 nm without any observable shift. There were no detected changes in the FWHM of the first exciton peak or the overall absorption coefficient. Similarly, PbSe QDs growth at a higher temperature of 210 °C exhibited no change in the absorption spectrum, even though it had a longer wavelength absorption peak of 1400 nm. (Figure 3c). The results concerning wavelength shifts indicate that pristine PbSe QDs show a rapid wavelength shift from the beginning of the test, whereas PalCl‐treated PbSe QDs exhibit no wavelength change. This demonstrates that even a small amount of 0.25 mmol is highly effective in passivating the surface of PbSe QDs (Figure 3d). We also assessed the thermal stability of the PbSe QDs under 120 °C conditions. Pristine PbSe QDs exhibited significant shifts in their absorption peak within 24 h (Figure S11a, Supporting Information). In contrast, the PalCl‐treated PbSe QDs demonstrated enhanced stability, with the 0.25 mmol treated QDs maintaining their absorption peak for 5 days (Figure S11b, Supporting Information) and the 0.5 mmol treated QDs sustaining their peak for 6 days (Figure S11c, Supporting Information). To further investigate this increased stability, we dispersed the synthesized PbSe QDs in TCE and exposed them to UV light to measure absorption changes. Similar to the solution stability tests, the PalCl‐treated PbSe QDs exhibited slight wavelength shifts but demonstrated significantly higher stability compared to pristine QDs (Figure S12a,b, Supporting Information). Furthermore, the same PalCl‐treated PbSe QDs, coated as a film on a glass substrate with TBAI treatment, also showed excellent stability (Figure S12c, Supporting Information).

Additionally, we measured the PL of PalCl 0.25 mmol PbSe QDs grown at 170 and 210 °C, as well as pristine PbSe QDs under the same conditions. The PL peaks and FWHM were measured as follows: 126 and 186 nm for PalCl 0.25 mmol PbSe QDs (170 and 210 °C, respectively), and 238 nm for pristine PbSe QDs (170 °C).(Figure S13, Supporting Information) For pristine PbSe QDs grown at 210 °C, the exact peak could not be determined due to measurement limitations. The results show that similar to the absorption spectrum, the PL of PalCl‐assisted PbSe QDs exhibits narrower FWHM compared to pristine PbSe QDs.

**Figure 4:**
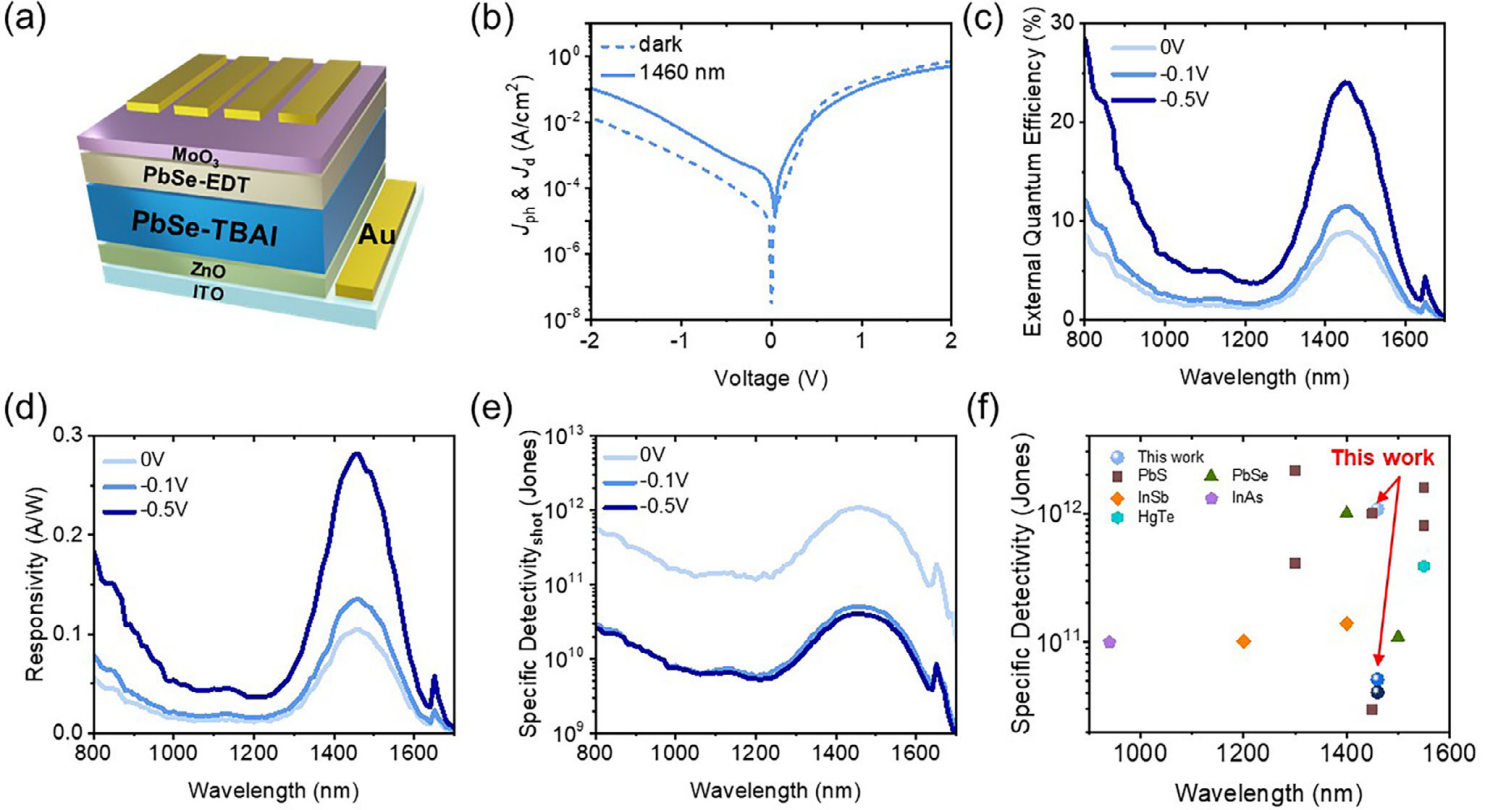
a) Device structure, b) *J*–*V* curve, c) external quantum efficiency, d) spectral responsivity, and e) specific de‐tectivity of photodetectors using PbSe QDs. f) Comparison of specific detectivities of reported QD‐based PDs.

Utilizing the synthesized PalCl‐treated PbSe QDs, we fabricated SWIR PDs and characterized their PD properties. The detailed fabrication method is described in the Experimental Section. We dispersed 0.5 mmol PalCl‐treated PbSe QDs in cyclohexane and used tetrabutylammonium iodide (TBAI) and 1,2‐ethanedithiol (EDT) to form PbSe‐TBAI and PbSe‐EDT as the active and hole extraction layers, respectively.^[^ ^35^, ^36^, ^37^ ^]^ As shown in **Figure** **4**, the device exhibited excellent specific detectivity (D^*^) of 4.08 × 10^10^ Jones at 1460 nm under the reverse bias of −0.5 V due to high responsivity (EQE) of 0.282 A W^−1^ (23.96%) at the corresponding wavelength (c.f. dark current density (J_d_: 1.49 × 10^−4^ A cm^−2^ at −0.5 V). Notably, the obtained D^*^ values at 0 and −0.1 V were 1.08 × 10^12^ and 5.14 × 10^10^ Jones, respectively, which are among the highest values for reported >1400 nm QD PD devices (Figure 4f).^[^ ^19^, ^38^, ^39^, ^40^, ^41^, ^42^, ^43^, ^44^, ^45^, ^46^, ^47^, ^48^ ^]^

Furthermore, we conducted a stability evaluation of the fabricated device. By storing the device in ambient conditions and evaluating its stability over 200 h, we confirmed that the device's performance was well‐maintained throughout the 200‐h period (Figure S14, Supporting Information). Additionally, to gain deeper insights into the fabricated device, we performed measurements of noise current,‐3 dB frequency, and response time. (Figure S15, Supporting Information). The noise spectral density measurements showed that at −0.1 and −0.5 V, the noise gradually approached the shot noise limit calculated based on dark current as the frequency increased. We obtained values of 1.21 × 10^10^ Jones at −0.1 V and 8.17 × 10^9^ Jones at −0.5 V. These values are lower than the shot noise‐based detectivity, as they account for total noise, including not only shot noise but also flicker noise and thermal noise. Using a 980 nm laser, the‐3 dB frequency was determined to be ≈25.67 kHz. Through response time measurements, we obtained rise and decay times of 1.20 × 10^−5^ s and 1.18 × 10^−5^ s, respectively.

Additionally, to analyze the effect of the quantum dot absorption layer thickness on device performance, devices with varying quantum dot thickness were fabricated and measured. The dark current showed little variation with changes in thickness, which was also reflected in the noise current, exhibiting a similar trend. In terms of EQE, a distinct peak was observed for the thinnest condition. Finally, detectivity calculations revealed that the device with the thinnest layer, where the EQE peak was most prominent, exhibited the highest detectivity (Figure S16, Supporting Information). The EQE peaks were not clearly resolvable in pristine PbSe‐based QD PDs due to the surface oxidation‐induced traps. (Figure 1c)^[^ ^39^, ^49^ ^]^ In the contrary, sharp absorption and strong EQE peak of PalCl‐treated PbSe in the SWIR regime further support the surface passivation effect of Cl^−^ for PbSe QDs. We also note that energy level alignment (Figure S17, Supporting Information) by employing bilayered PbSe‐TBAI and PbSe‐EDT further facilitated efficient charge transport in trap‐passivated PalCl‐treated PbSe PDs.^[^ ^43^ ^]^

## 3 Conclusion

In this study, we successfully functionalized the surface of QDs with chloride ions (Cl^−^) by introducing a minimal amount of palmitoyl chloride during the synthesis process. From the perspective of quantum dot synthesis, surface passivation with halide ions, such as Cl^−^, is of critical importance. This approach is particularly effective not only for Pb‐chalcogenide QDs but also for pnictogen‐based QDs, II‐VI, and ternary QDs, where halide ligands serve as robust surface passivants. Ligands such as primary amines or phosphines, which are L‐type ligands, exhibit relatively weak binding affinity, leading to suboptimal surface passivation. Stronger ligands, such as carboxylic acids, while offering enhanced binding, pose the risk of inducing oxidation. In contrast, halides are monatomic and possess high electronegativity, which confers strong binding affinity and superior surface passivation properties compared to other ligand types. Conventional halide passivation strategies typically involve the use of oleylamine (OLA) or trioctylphosphine (TOP) during the synthesis process to introduce halides onto the quantum dot surface. However, the addition of oleic acid (OA) is often necessary to maintain dispersibility and colloidal stability. Alternatively, post‐synthetic treatments using precursors such as metal halides or ammonium halides are also employed. Our method, however, enables effective Cl^−^ incorporation in a single synthetic step without the need for additional post‐synthetic treatments, achieving significant passivation efficiency with as little as 5% of the total ligand composition. Given the mechanistic insights into our acidic chloride‐mediated synthesis approach, we believe this method holds substantial potential for broader application across various quantum dot synthesis protocols. Thus, we propose this method as a viable strategy for enhancing the stability and optical performance of QDs.

## 4 Experimental Section

### Materials

All chemicals, Lead acetate trihydrate (Pb(OAc)_2_•3H_2_O, 99.99% trace metal basis, Sigma–Aldrich), oleic acid (OA, 90% technical grade, Aldrich), palmitoyl chloride (PalCl, 98%, Aldrich), 1‐octadecene (ODE, 90%, Aldrich) hexane (anhydrous, Sigma–Aldrich), tetrabutylammonium iodide (TBAI, 99.99%, Aldrich), 1,2‐ethandithiol (EDT, 99%, Aldrich), methanol (MeOH, anhydrous, Aldrich), acetonitrile (ACN, anhydrous, Aldrich), tetrachloroethylene (TCE, anhydrous, Sigma–Aldrich), tri‐n‐octylphosphine (TOP, 97%, Strem), bis(trimethylsilyl)selenium (TMS‐Se, 99%, JSI‐silicon) were used without any further purification.

### Synthesis of PbSe QDs

The PbSe QDs were prepared according to S. Jeong's report with slight modifications.33 To prepare the Se precursor solution, 0.2 mL of TMS‐Se (1 mmol) and 1 mL of TOP in a vial were mixed at room temperature in a nitrogen‐filled glove box. For pristine PbSe QDs, 0.949 g of Pb(OAc)_2_•3H_2_O (2.4 mmol) and 1.6 mL of OA (4.8 mmol) were loaded in a 50 mL 3‐neck‐flask with vigorous stirring, and then the mixture was degassed under vacuum degassed for 10 min at RT. After degassing, the Pb‐oleate solution was heated to 110 °C under the N_2_ atmosphere using an outgassing flow. After 1 h, the TMS‐Se solution was quickly injected into the Pb‐oleate solution at 85 °C under a nitrogen atmosphere, the mixture was stirred at the target temperature (170–280 °C) for 10 min, and then cooled to room temperature. For PalCl‐treated PbSe QDs, the synthesis was carried out using the same procedure as for Pb‐oleate, except the quantity of oleic acid (OA) is reduced by the amount of PalCl introduced during the Pb‐oleate preparation stage. PbSe QDs were precipitated from the reaction by adding ethanol, acetone, and hexane mix solvent (volume fraction 1:1:1.2) and separated by centrifugation.

### Device Fabrication

The ITO/glass substrates were cleaned using detergent, deionized water, acetone, and isopropanol in a sonicator for 15 min for each, followed by UV‐ozone treatment for 10 min. Then, the ZnO precursor was prepared in 1 mL of 2‐methoxyethanol using 0.1 g of Zn(OAc)_2_ dehydrate and 0.028 mg of ethanolamine. The solution was spin‐coated onto the cleaned substrates and then annealed at 200 °C for 20 min. The prepared QD solution was spin‐coated onto ZnO films. For PbSe‐TBAI layers, PbSe QD solution was dispersed in cyclohexane (15 mg mL^−1^) and spin‐cast at 2500 rpm for 30s. Then, ligand exchange was performed with TBAI solution (20 mg mL^−1^ in MeOH) by spin‐coating s and rinsing with MeOH. For PbSe‐EDT layers, PbSe QD solution was dispersed in cyclohexane (15 mg mL^−1^) and spin‐cast at 2500 rpm for 30s. Then, ligand exchange was performed with EDT solution (0.5vol.% in ACN) by spin‐coating s and rinsing with ACN. All spin‐coating processes were conducted under a dry N_2_ environment in a glove box. Finally, MoO_3_ (9 nm) and Au (100 nm) layers were thermally deposited in sequential using shadow masks for electrode patterning under a vacuum pressure of 2.0 × 10^−6^ Torr.

### Characterization Procedures

Absorption spectra were measured using a SCINCO PDA S‐3100 UV–vis spectrophotometer. Emission spectra were measured using a JASCO FP‐6500 fluorescence spectrometer. The nanoparticles were dispersed in hexane and spread on a copper grid and silicon wafer for these measurements. TEM images were collected using an FEI Tecnai TITAN 80–300TM transmission electron microscope with an acceleration voltage of 150 kV. High‐resolution TEM images were obtained on an FEI Titan microscope operated at 300 kV. SEM images and X‐ray mapping data were obtained using a JSM‐6700F field emission scanning electron microscope equipped with an INCA energy dispersive X‐ray spectrometer at an operating voltage of 30 kV.

### Electrical Characterization of the QD‐PDs

The *J*–*V* curves were obtained using a semiconductor analyzer (Keithley 4200‐SCS) under dark conditions. Spectral R values were obtained from the EQE spectra of the devices, which were measured using an incident photon‐to‐current conversion efficiency system consisting of an Xe lamp (Newport, 100 W) and a monochromator. The UV–vis absorption spectra of the QD films were measured using a UV–vis spectrophotometer (Jasco, V‐770).

## Conflict of Interest

The authors declare no conflict of interest.

## Author Contributions

H.S. and A.Y.L. contributed equally to this work.

## Supporting information



Supporting Information

## Data Availability

The data that support the findings of this study are available from the corresponding author upon reasonable request.
