# Supplementary material for: High‐Performance PbSe Quantum Dots with Palmitoyl Chloride and Their Application to Short‐Wavelength Infrared Photodetector Devices
Source: Small Methods. 2025 Mar 12;9(8):2402237. doi: 10.1002/smtd.202402237 (PMC12391623; doi:10.1002/smtd.202402237)
Supplement: Supplementary file 1 — Supporting Information [file SMTD-9-2402237-s001.docx]

Supporting Information

High-Performance PbSe Quantum Dots with Palmitoyl Chloride and Their Application to Short-Wavelength Infrared Photodetector Devices

Haewoon Seo,^a,b,ǂ^ Ah Young Lee,^aǂ^ Eun Hye Lee,^a^ Dong Won Kim,^a,b^ Hyo Jin Hwang,^a^ Sunghoon Kim,^c^ Jong H. Kim,^*a^ Sang-Wook Kim^*a^

^a^AI-Superconvergence KIURI Translational Research center, Ajou University, Suwon 443-749, Republic of Korea

^b^Department of Molecular Science and Technology, Ajou University, Suwon 443-749, Republic of Korea

^c^Department of Applied Chemistry, Dong-Eui University, Busan 47340, Republic of Korea

^*^Email: jonghkmin@ajou.ac.kr, swkim@ajou.ac.kr

**
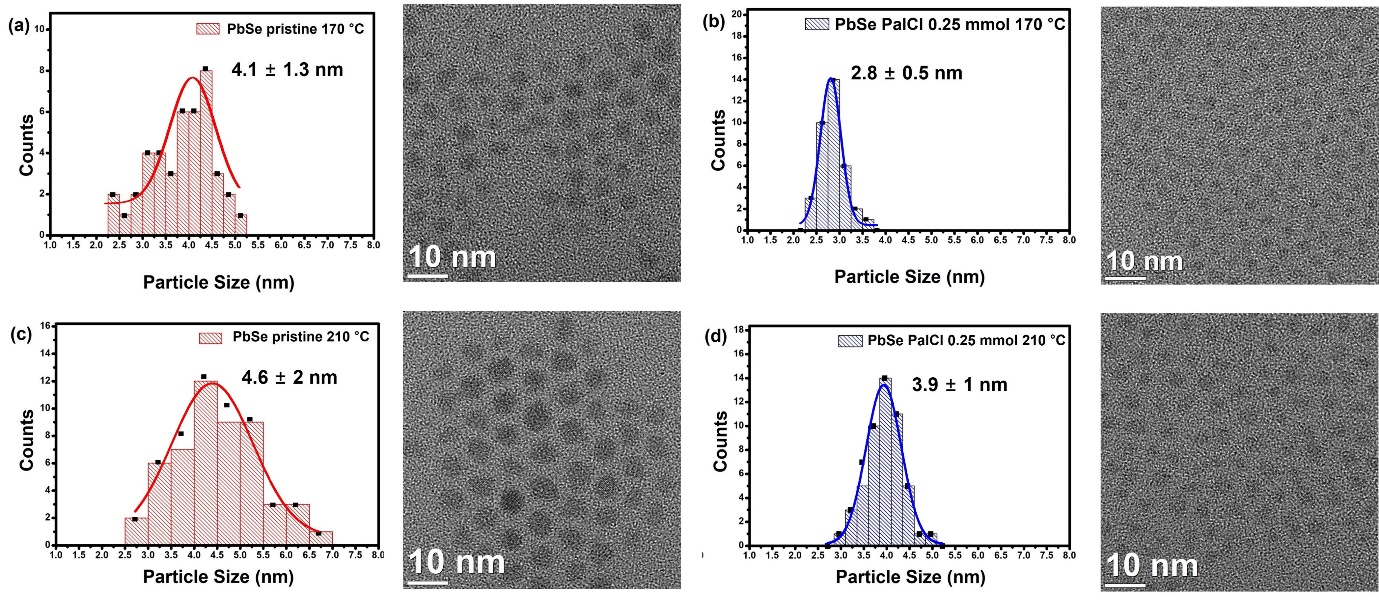
****Figure S1**. Size distribution data of PbSe QDs from the TEM images in Figure 1. (a,b) PbSe pristine QDs growth at 170 °C, (c,d) PalCl 0.25 mmol treated PbSe QDs growth at 170 °C**,** (e,f) PbSe pristine QDs growth at 210 °C, (g,h) PalCl 0.25 mmol treated PbSe QDs growth at 210 °C.


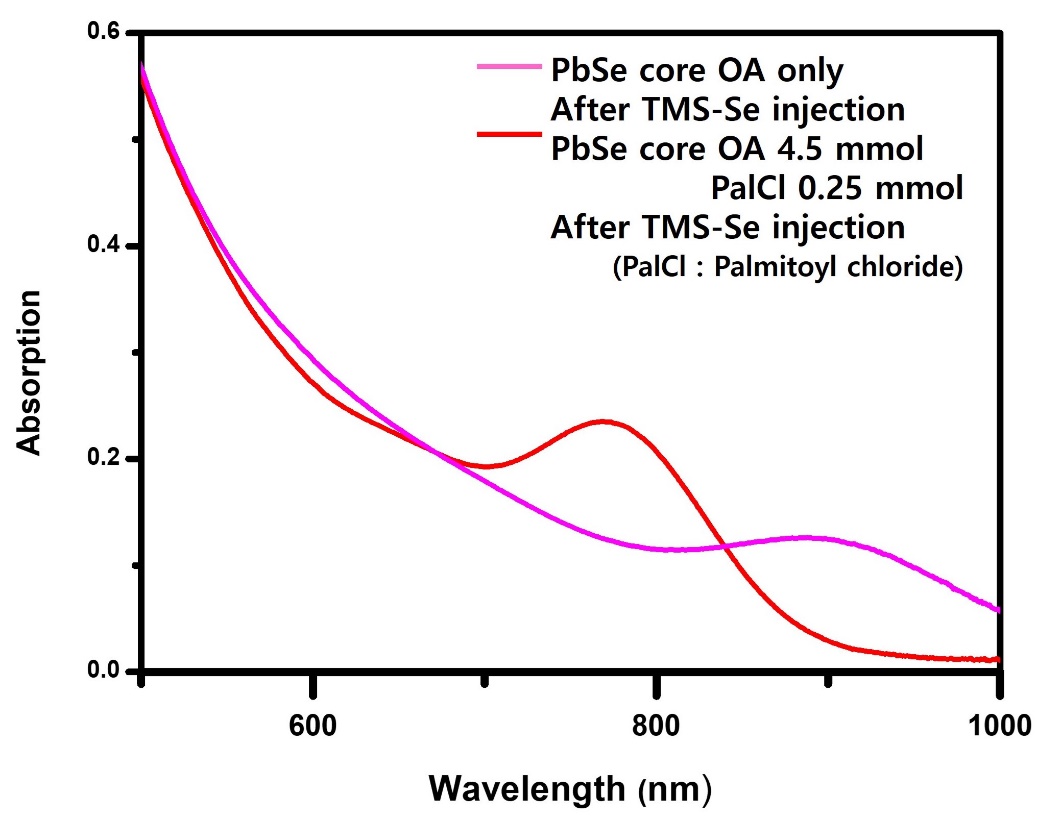
**Figure S2**. Absorption spectrum of PbSe QDs after the injection of the Se precursor. (Red line : PalCl 0.25 mmol treated / pink line : pristine).

**
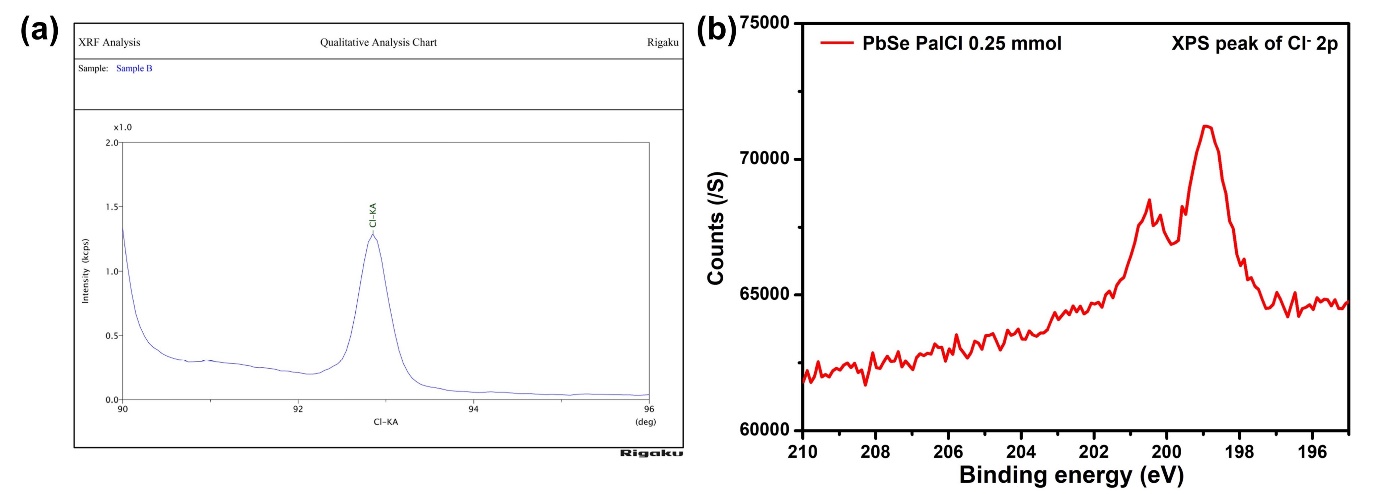
Figure S3.** (a) X-Ray Fluorescence spectroscopy (XRF) spectrum data and X-ray Photoelectron Spectroscopy (XPS) of Cl^-^ for the PalCl 0.25 mmol treated PbSe QDs.


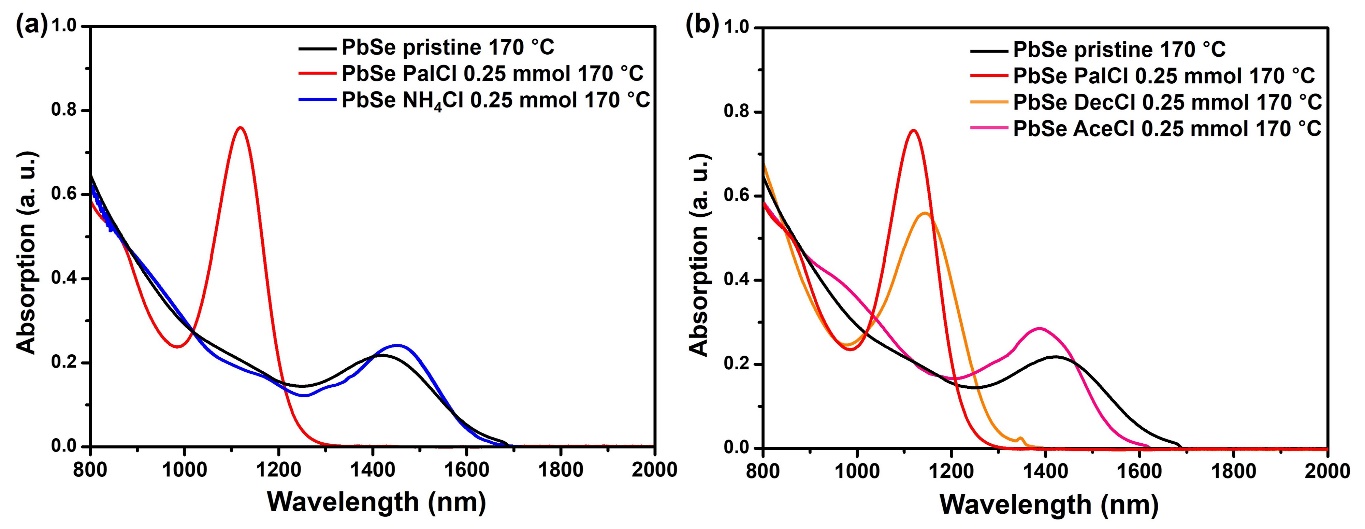


**Figure S4**. Comparison of absorption data between PalCl 0.25 mmol treated PbSe QDs, pristine PbSe QDs, and other Cl⁻ sources. (a) Comparison of absorption spectra with ammonium chloride (NH_4_Cl). (b) Comparison of absorption spectra with decanoyl chloride (DecCl) and acetyl chloride (AceCl).

**
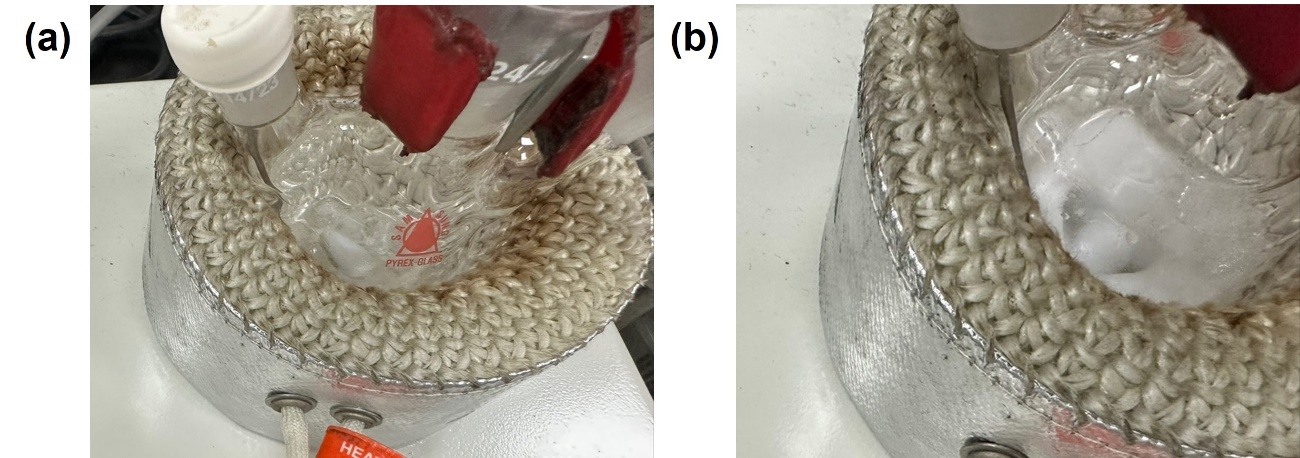
Figure S5.** Photos of the solutions during the Pb-oleate preparation stage, comparing with and without PalCl treatment. (a) No PalCl. (b) PalCl 0.25 mmol added.

**
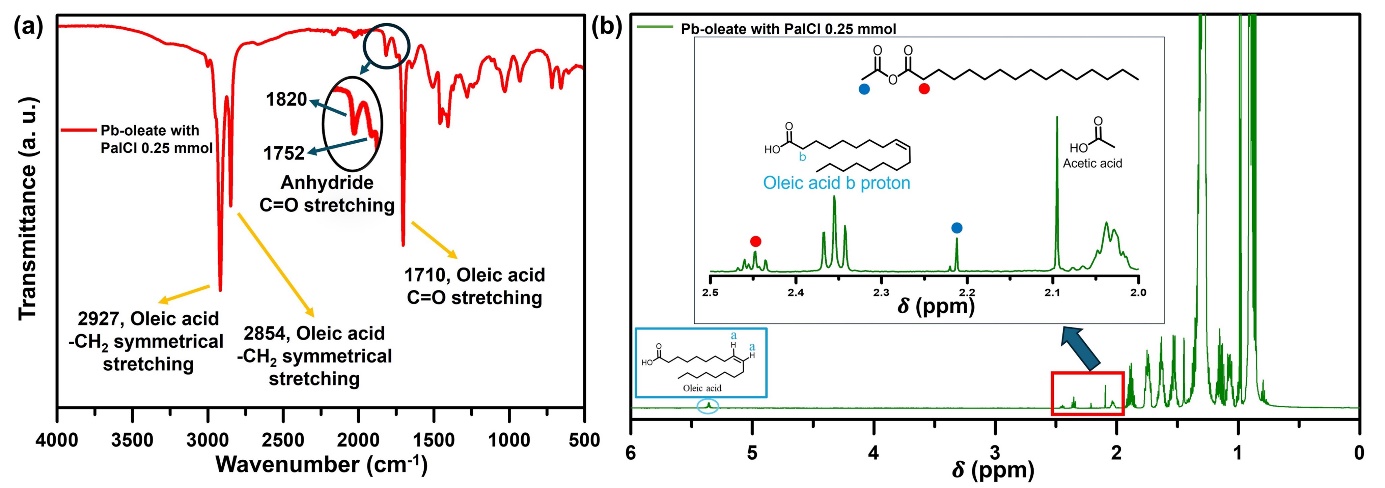
**

**Figure S6**. Analysis data of Pb-oleate with the addition of 0.25 mmol of PalCl. The formed Pb-oleate was centrifuged, and the supernatant was measured. (a) FT-IR, (b) ^1^H-NMR.

**
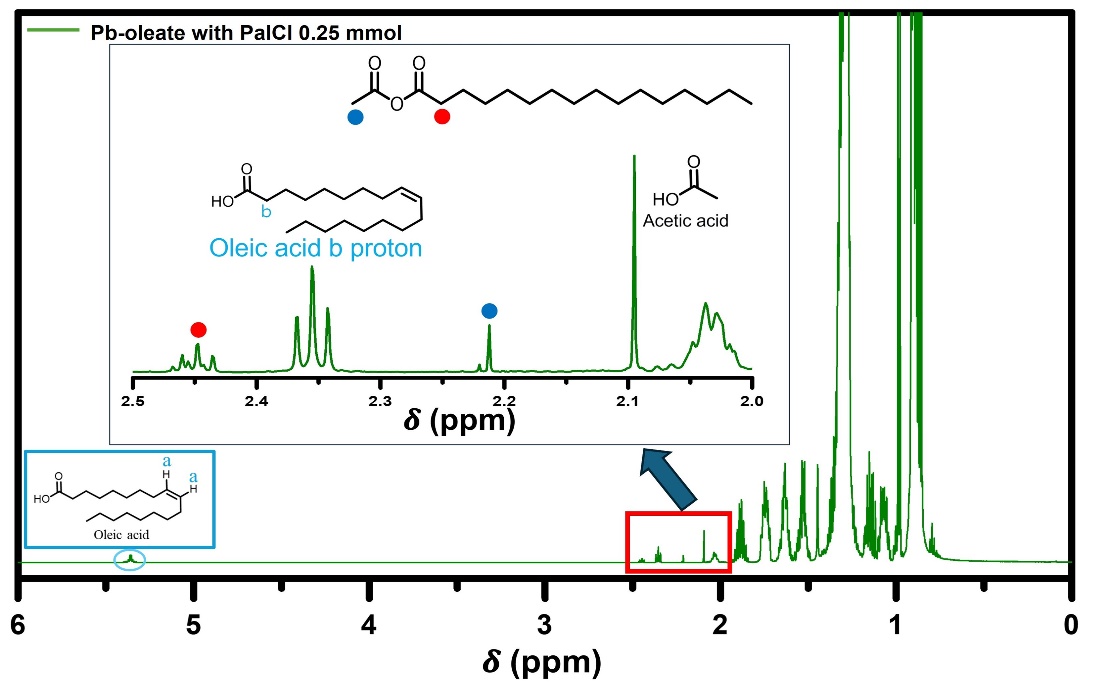
**

**Figure S7.** The ^1^H-NMR spectrum for the PalCl 0.25 mmol treated PbSe QDs in the 2.3 - 2.5 ppm region.

**
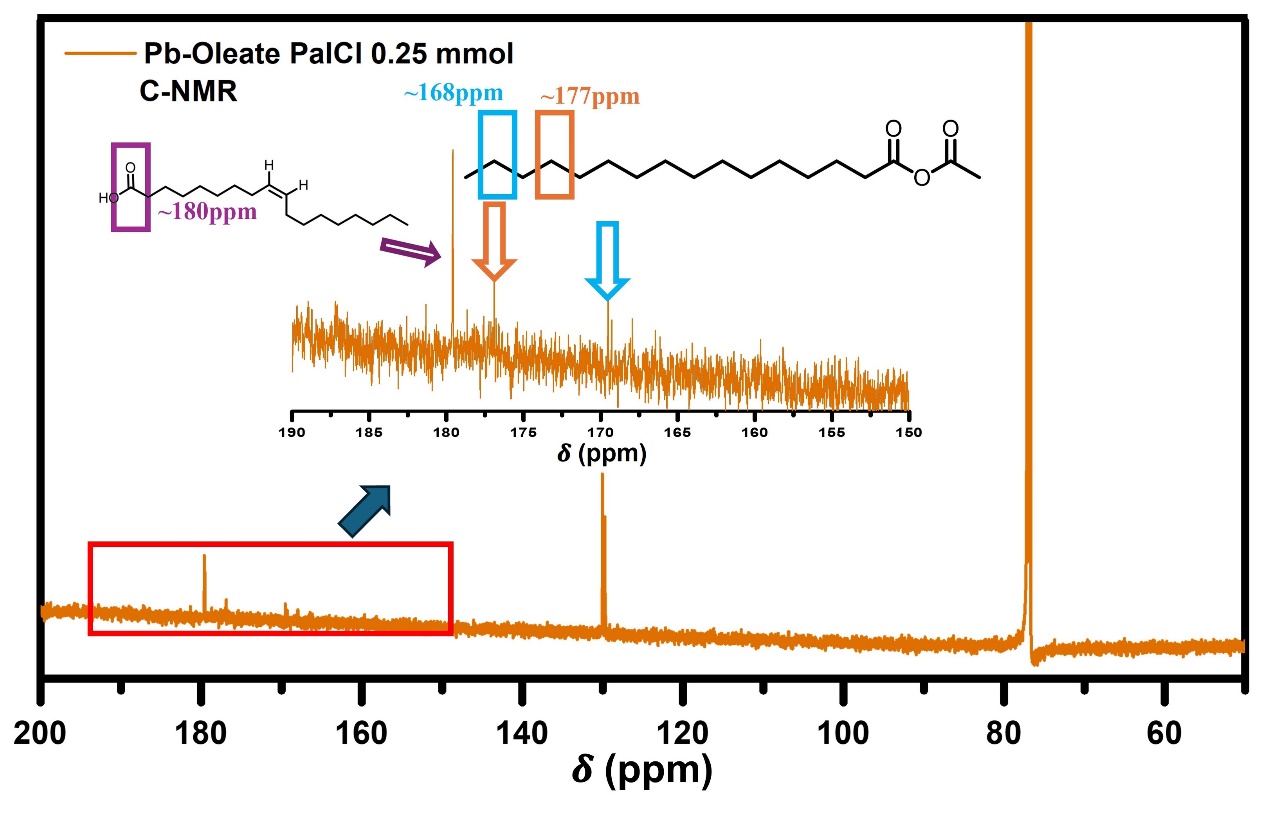
**

**Figure S8.** The ^13^C-NMR spectrum for the PalCl 0.25 mmol treated PbSe QDs.

**
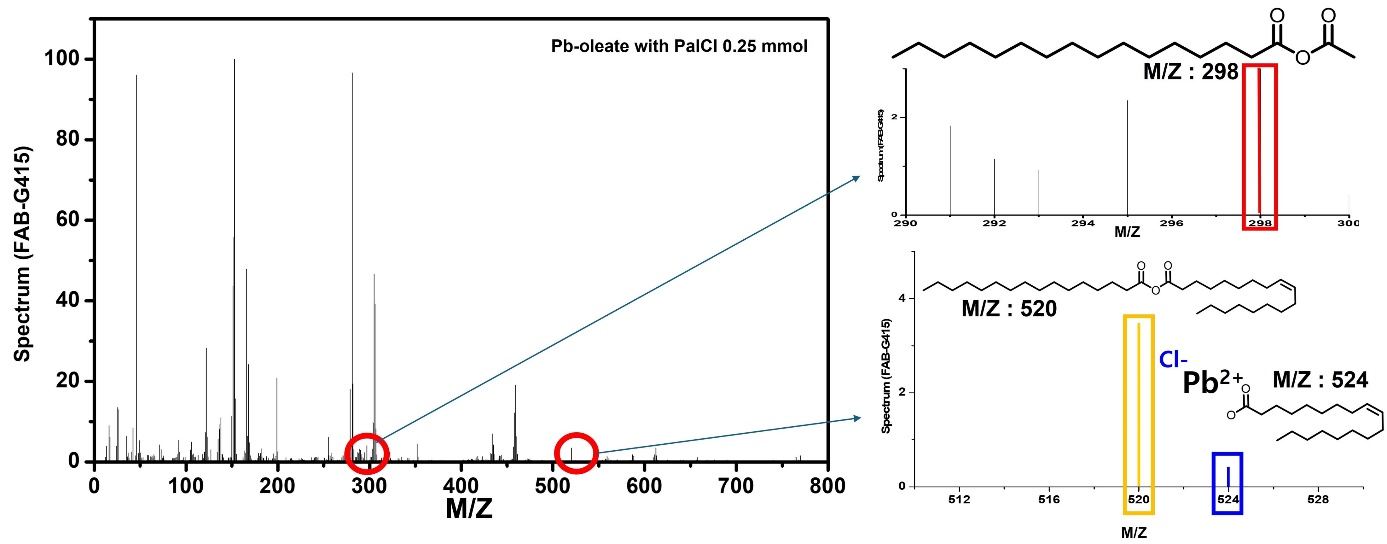
**

**Figure S9.** FAB-Mass spectrum of Pb-oleate with the addition of 0.25 mmol of PalCl. The formed Pb-oleate was centrifuged, and the supernatant was measured.

**
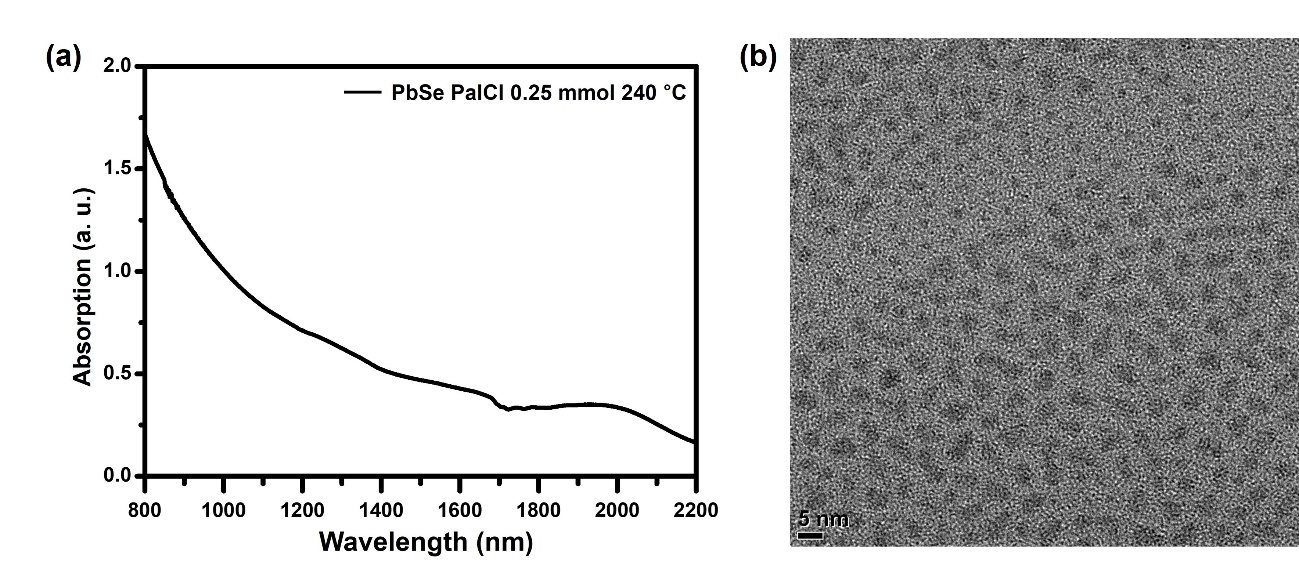
Figure S10**. Data of PalCl 0.25 mmol treated PbSe QDs growth at 240 °C. (a) Absorption spectrum, (b) TEM image.

**
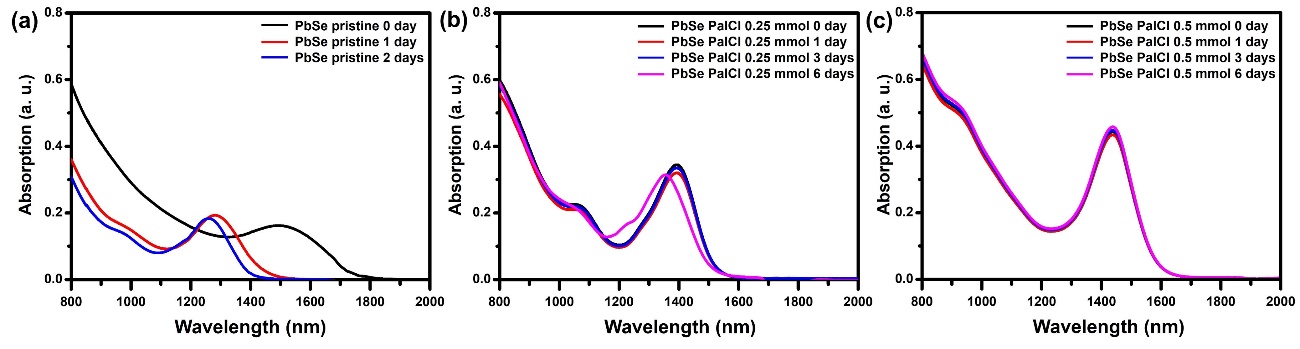
Figure S11.** Comparing time-dependent changes in the absorption spectrum of PbSe QDs in 120 °C environment. (a) Pristine PbSe QDs growth at 170 °C. (b) 0.25 mmol of PalCl treated PbSe QDs growth at 210 °C. (c) 0.5 mmol of PalCl treated PbSe QDs growth at 230 °C.


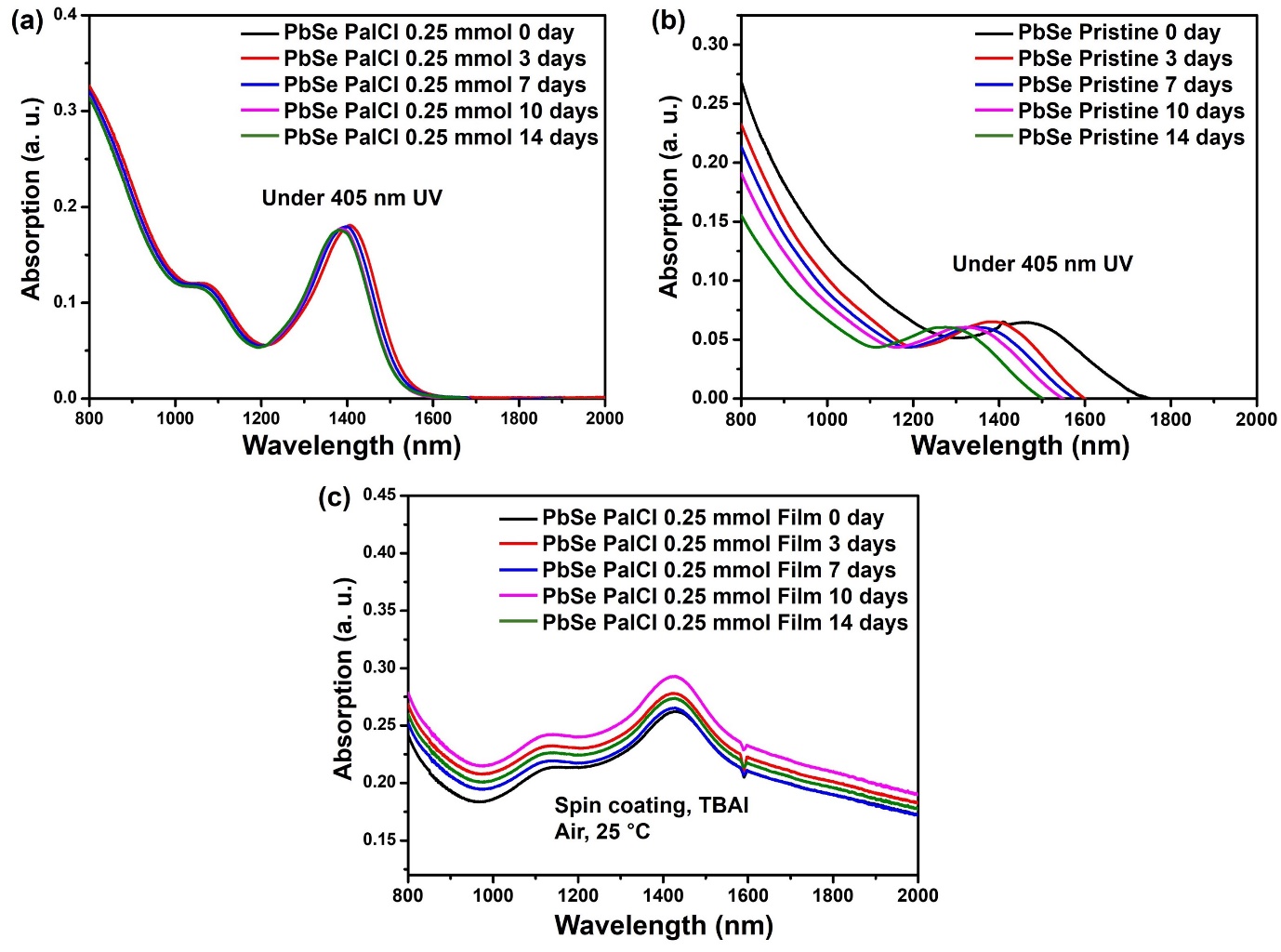


**Figure S12**. Comparing time-dependent changes in the absorption spectrum of PbSe QDs. (a) 0.25 mmol of PalCl treated PbSe QDs solution under UV light. (b) Pristine PbSe QDs under UV light. (c) 0.25 mmol of PalCl treated PbSe QDs film.


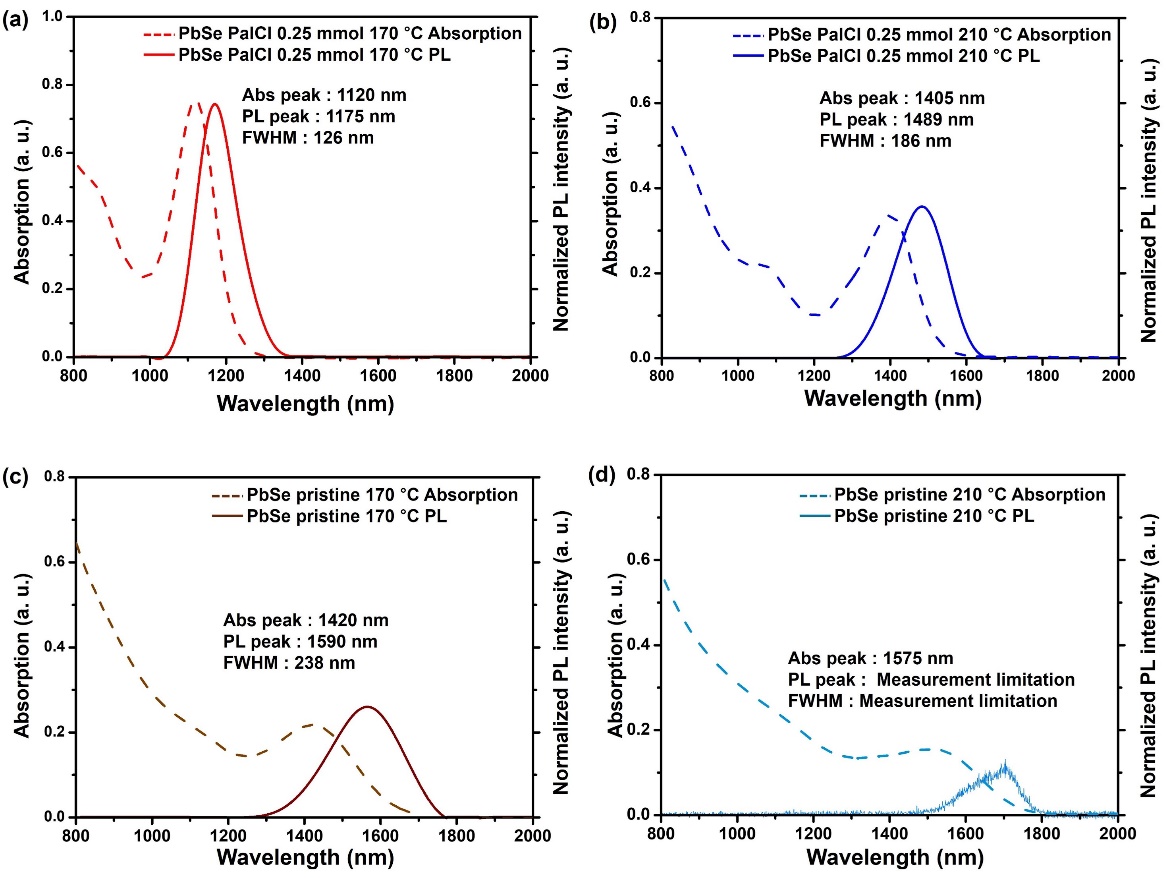


**Figure S13.**  Absorption and PL spectrum data of PbSe QDs with and without PalCl addition. PalCl 0,25 mmol treated PbSe QDs (a) 170 °C growth, (b) 210 °C growth. Pristine PbSe QDs (c) 170 °C growth, (b) 210 °C growth.


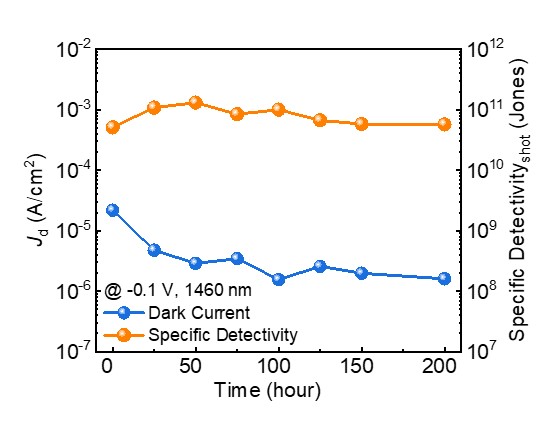


**Figure S14.** Device stability of dark current and specific detectivity of SWIR PbSe QD PD during storage in the ambient condition for 200 h.


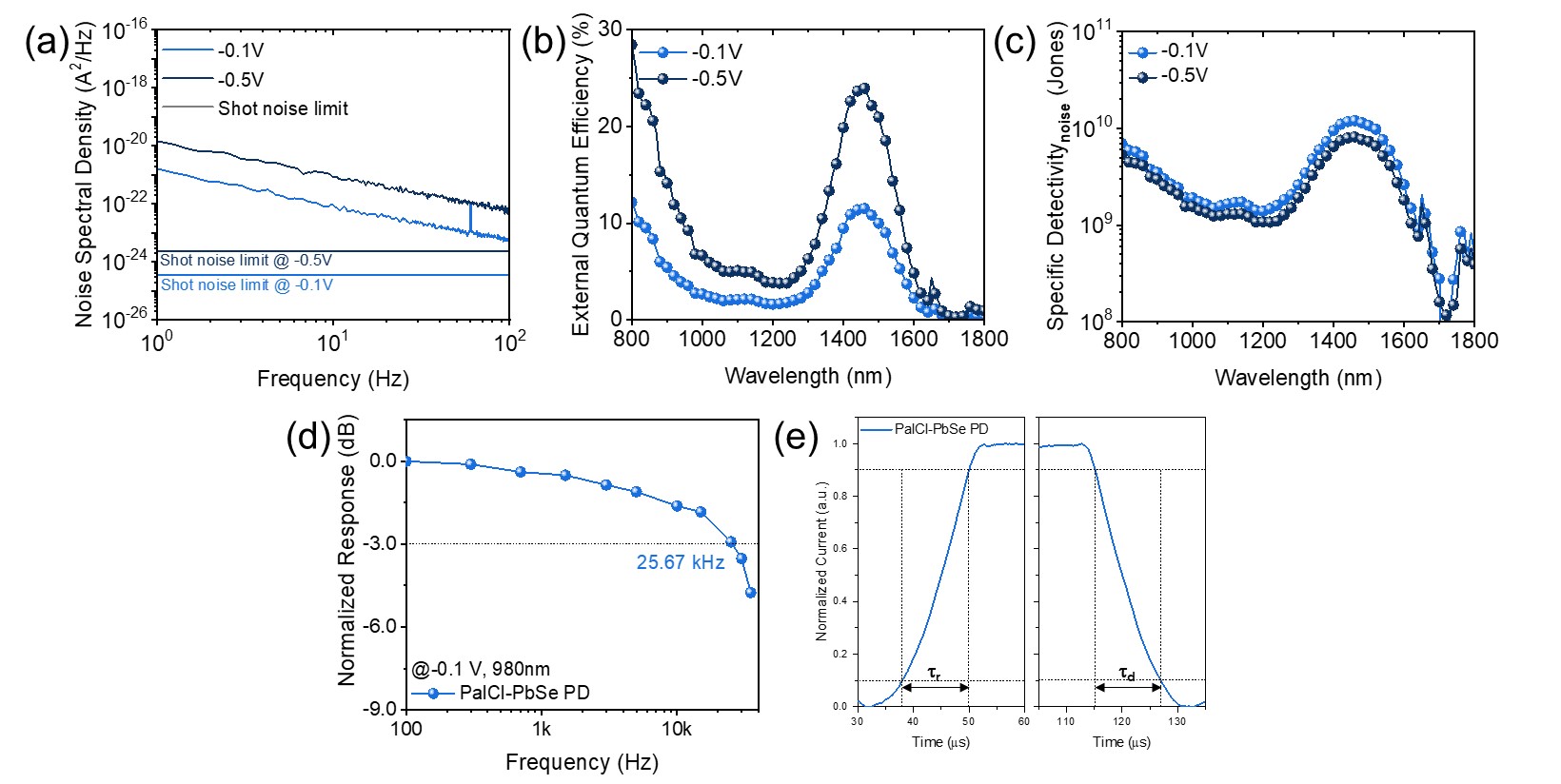


**Figure S15.** Device performance of SWIR PbSe QD PDs. (a) noise spectral density, (b) external quantum efficiency, (c) specific detectivity based on spectral noise density, (d) -3dB frequency, (e) response time.


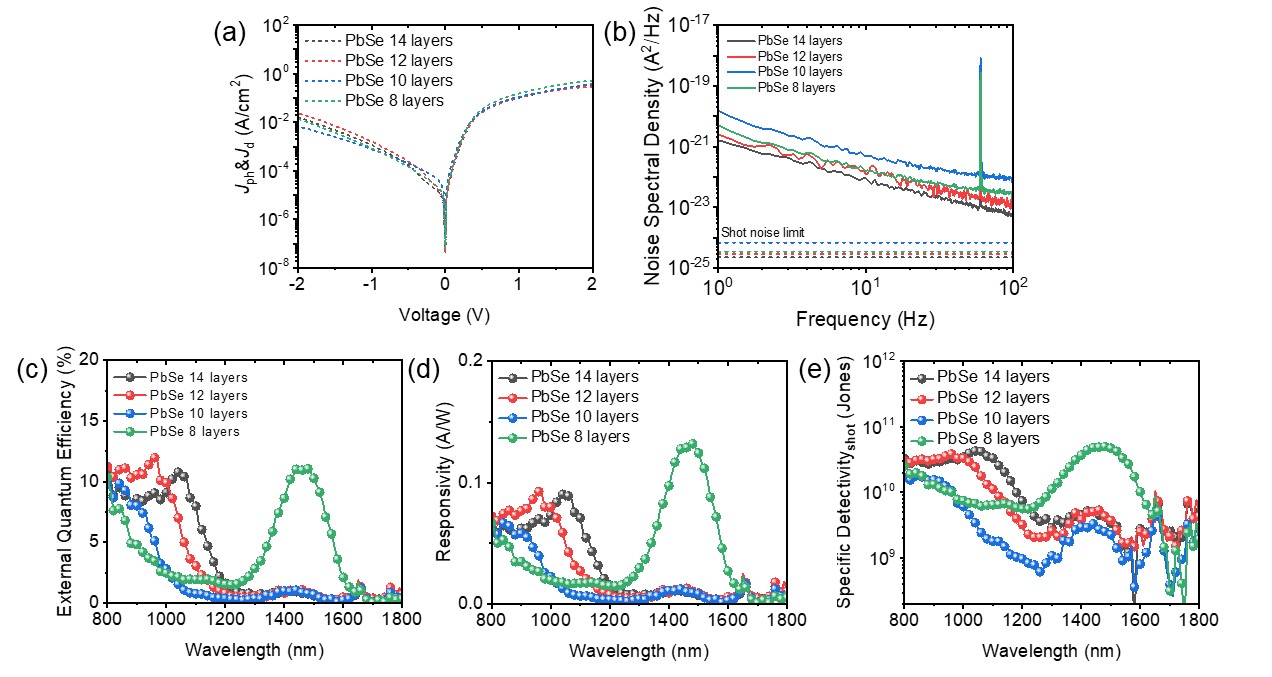


**Figure S16.** Device performance with varying the thickness of the QD layer. (a) J-V curve, (b) noise spectral density, (c) external quantum efficiency, (d) responsivity, and (e) specific detectivity.


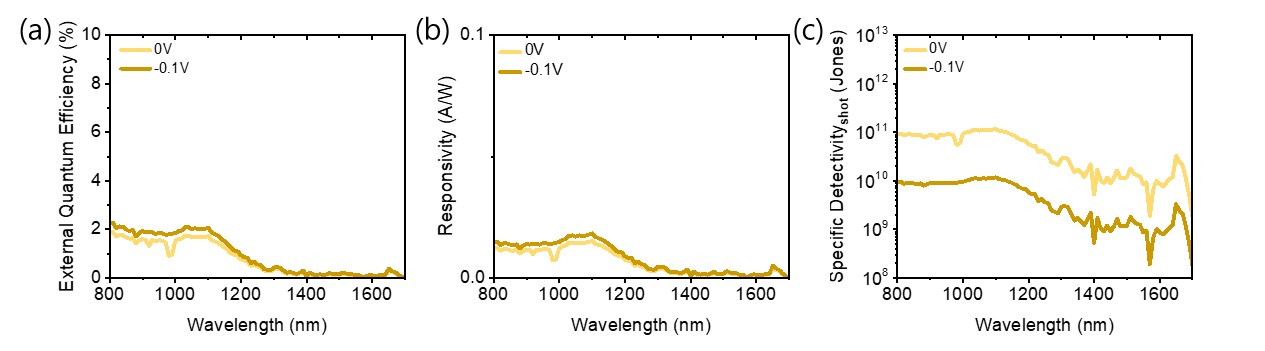


**Figure S17.**  Device performance of PbSe pristine-based QD PD. (a) EQE, (b) responsivity, and (c) specific detectivity based on the shot noise.
